# Mitochondrial sirtuin 4 shapes the intestinal microbiota of *Drosophila* by controlling lysozyme expression

**DOI:** 10.1186/s42523-025-00431-x

**Published:** 2025-06-13

**Authors:** Mirjam Knop, Christian Treitz, Stina Bettendorf, Judith Bossen, Jakob von Frieling, Shauni Doms, Abdulgawaad Saboukh, Iris Bruchhaus, Ronald P. Kühnlein, John F. Baines, Andreas Tholey, Thomas Roeder

**Affiliations:** 1 Department Zoology, Kiel University, Kiel, Germany; 2 IEM, Systematic Proteomics, Kiel University, Kiel, Germany; 3 IEM, Section of Evolutionary Medicine, Kiel University, Kiel, Germany; 4 Bernhard Nocht Institute for Tropical Medicine, Hamburg, Germany; 5 Institute of Molecular Biosciences, University of Graz, Graz, Austria; 6 BioTechMed-Graz, Graz, Austria; 7 Field of Excellence BioHealth, University of Graz, Graz, Austria; 8 Max Planck Institute for Evolutionary Biology, Plön, Germany; 9 German Center for Lung Research (DZL) - Airway Research Center North (ARCN), Kiel, Germany; 10 CAU Kiel, Zoology, Olshausenstrasse 40, 24098 Kiel, Germany

## Abstract

**Background:**

Sirtuins are deacetylases that are highly conserved throughout the animal kingdom. They act as metabolic sensors that coordinate cellular responses, allowing an adapted response to various stressors. Epithelial cells, especially those of the intestine, are directly exposed to a wide range of stressors. Together with the microbiota, they form a complex ecosystem with mutual influences. The significance of sirtuins in this complex system is still waiting to be clarified.

**Results:**

Here, we show that a protein-restricted diet strongly increases the intestinal expression of sirtuin 4 (*dSirt4*), the only mitochondrial sirtuin in *Drosophila*. To elucidate the effects of deregulated *dSirt4* expression in the intestine, we analyzed *dSirt4* knockout flies. These flies showed substantial changes in their intestinal proteome and physiological properties. One of the most striking effects was the strong induction of lysozymes in the intestine, with a corresponding increase in lysozyme activity. This effect was organ-autonomous, as it was also observed in flies with *dSirt4* knocked out only in intestinal enterocytes. The significant increase in lysozyme abundance in response to tissue-specific *dSirt4* knockdown did not reduce the total number of bacteria in the intestine. However, it did affect the microbiota composition by reducing the number of gram-positive bacteria. This effect on microbiota composition can be attributed to *dSirt4*-dependent lysozyme expression, which is absent in a lysozyme-deficient background. *dSirt4* knockout in the enterocytes shortened the lifespan of the flies, as did ectopic lysozyme overexpression in the enterocytes.

**Conclusions:**

The only mitochondrial sirtuin in *Drosophila*, *dSirt4*, is induced by dietary stress in intestinal epithelial cells, which directly regulates the lysozyme activity of these cells. We could associate this altered lysozyme activity with a shift in the microbiota composition, demonstrating a direct link between stress, nutrition, and the host’s microbiota regulation.

**Supplementary Information:**

The online version contains supplementary material available at 10.1186/s42523-025-00431-x.

## Introduction

The intestinal epithelium is a central metabolic organ that orchestrates various metabolic functions. Despite its seemingly simple architecture, the intestinal epithelium is characterized by a marked cellular complexity that appears to be phylogenetically conserved since it is observed throughout the animal kingdom [1, 2]. The intestinal epithelium and the endogenous microbiota are constantly exposed to diverse environmental factors [3, 4], the most important of which is the diet, which varies greatly in energy content and quality [5]. Therefore, one of the main functions of the intestinal epithelium, with the closely associated microbiota, is maintaining homeostasis under these changing environmental conditions [6]. Disrupting this homeostatic balance is causally linked with dysbiosis and intestinal diseases, including inflammatory bowel diseases (IBDs) [7, 8].

To adapt to changing conditions, intestinal cells require cellular sensors. Sirtuins, nicotinamide adenine dinucleotide (NAD^+^)-dependent protein deacetylases, can fulfill this role [9]. They are a group of highly conserved proteins sharing homology with the yeast silent information regulator 2 (Sir2) [10] that are involved in diverse biological processes, including metabolism, aging, DNA repair, and regulating microbiota [11, 12]. They help to maintain functional and structural integrity in all organs [13]. The observation that sirtuin activity is required to maintain intestinal integrity, even in planarians, implies that this protein family has a phylogenetically ancient role in maintaining organ homeostasis [14].

Studies using mice with gut enterocytes deficient in sirtuin 1 (SIRT1) showed that SIRT1 is required to prevent IBD development. This effect seems to be mediated by shaping the gut microbiota [15], and SIRT1 activity is required to prevent the translocation of harmful bacteria from the intestinal lumen into the bloodstream [15]. SIRT1-deficient intestines have more goblet and Paneth cells, which changes the antimicrobial tone in the intestinal lumen and can affect the gut microbiota composition [16]. SIRT1 levels are also reduced in biopsies from patients with IBD [17]. Moreover, SIRT1 is required for bile acid absorption because it directly targets the HNF1 homeobox A (HNF1α)/farnesoid X receptor (FXR) signaling pathways [18]. In addition to the effects of SIRT1 on microbiota composition, especially beneficial members of the microbiota can upregulate the intestinal expression of *SIRT1* [19], demonstrating the strong interconnection between sirtuin signaling and the microbiota.

A subgroup within the sirtuin family, the mitochondrial sirtuins—sirtuins 3 (SIRT3), 4 (SIRT4), and 5 (SIRT5)—have attracted particular interest because they act directly in the energy centers of the cell [20, 21]. Their crucial role in metabolic control, resulting from this activity in mitochondria, is particularly important because IBDs are associated with mitochondrial dysfunction [22, 23]. For example, dysfunctional *SIRT3* expression is associated with impaired gut barrier function, which is caused by dysbiosis, demonstrating the critical role of mitochondrial sirtuins in maintaining intestinal homeostasis [24]. Despite the studies that describe the pivotal role of sirtuins, especially mitochondrial sirtuins, in maintaining intestinal homeostasis, our mechanistic understanding of these processes is still rudimentary.

Simple models are helpful to better understand the general importance of mitochondrial sirtuins and their role in controling microbiota composition. *Drosophila melanogaster* only has one mitochondrial sirtuin, sirtuin 4 (*dSirt4*), which significantly influences lifespan; the modest fat body-targeted overexpression of this gene prolongs life. In contrast, *dSirt4* knockout flies have a shorter lifespan [25]. *dSirt4* plays an important role in the communication between cells and their mitochondria in a genotype-specific manner [26]. A connection between *dSirt4* and the microbiota was first demonstrated by the interaction between the endosymbiont *Wolbachia* and the expression level of *dSirt4* [27].

To gain a deeper mechanistic understanding of the processes controlled by mitochondrial sirtuins in the intestine, we turned to the fruit fly *Drosophila*. We found that some phenotypes observed in *dSirt4* knockout flies also occur in flies with *dSirt4* silenced only in intestinal cells. The most exciting finding was that knocking out *dSirt4* in enterocytes dramatically increases lysozyme activity, which directly affects microbiota composition.

## Materials and methods

### *Drosophila* stocks and culture

The following strains were obtained from the Bloomington *Drosophila* Stock Center (BDSC): *w*^*1118*^ (#5905), *dSirt4* KO (#8840), *UAS-Cas9* (#54592), and *Sirt4 sgRNA* (#78741). *Sirt4* KO flies were backcrossed to *w*^*1118*^ flies for several generations before experiments. *NP1-Gal4;tubPGal80ts* were kindly provided by D. Ferrandon and *Lys*^*B−PΔ*^ by Bruno Lemaitre. UAS-*sirt4* CRISPR/Cas9 was generated by combining *UAS-Cas9* and *Sirt4 sgRNA*. *UAS-lysB* and *UAS-lysP* were generated using the pBID-UASC vector [28] and *EcoR*I, *Bgl*II, and *BamH*I restriction enzymes. The cDNA of *lysB-PA* was amplified using the primers GAGAATTCCAAAATGAAGGCTTTCATCGTTCTG and GAGGATCCGAAGCAGTCATCGATGGAC primers, and *lysP* was amplified using the primers GAGAATTCCAAAATGAAAGCTTTTCTTGTGA and GAAGATCTGCAACTGTTGATCGAGGGCA. Flies were cultivated on a standard diet (NM; per 500 ml: 31.25 g brewer’s yeast, 31.25 g cornmeal, 10 g D-glucose, monohydrate, 5 g agar-agar, 15 g sugar beet syrup, 15 g molasses, 5 ml of propionic acid [10% in double distilled water], and 15 ml of nipagin [10% in 70% ethanol] for preservation). Temperature-sensitive crosses were raised at 18 °C; all others were raised at 25 °C. If not stated otherwise, 5–7-day-old mated female flies were used for the experiments. The F1 progeny with temperature-inducible genetic modules and their corresponding controls were kept at 18 °C for five days before we induced the expression of the gene of interest at 29 °C for five days. A holidic diet was used to induce protein malnutrition, as described earlier [29].

### Lifespan and infection survival time

The lifespan experiment was performed in standard *Drosophila* vials. Flies (females) were monitored daily and transferred onto fresh NM every 2–3 days until all the flies died. The lifespan of flies under starvation conditions experiment was performed in standard *Drosophila* vials filled with ~ 10 ml of 1.5% agar-agar to prevent them from dying of thirst. Dead flies were counted every two hours until all the flies died. The influence of DSS (MP Biochemicals, Canada) on the lifespan of *Drosophila* was tested in standard *Drosophila* vials filled with ~ 10 ml of 1.5% agar-agar. A solution of 5% DSS (w/v) in 5% sucrose (w/v) was applied to filter paper strips. The filter paper was exchanged every two days and the vials once a week. Dead and escaped flies were counted daily, and sucrose controls were counted every second day.

To measure survival after infection, overnight cultures of *Serratia marcescens* (Db11) were grown at 30 °C in an LB medium supplemented with streptomycin (10 µg/ml). Cultures were concentrated by centrifugation and resuspended in 5% sucrose to an optical density at 600 nm (OD_600_) of 50. The bacterial solution was applied on filter paper strips and the filter paper was changed every two days. Controls were fed with 5% sucrose only.

### Food consumption assay

Food consumption was assessed using a previously described consumption-excretion method [30] with minor modifications. NM or blue-dyed NM (0.5% [w/v] Brilliant Blue FCF) was pipetted into the caps of 2 ml screw cap vials. Individual flies were transferred into 2 ml screw cap vials with regular NM and the vials were loosely closed to ensure an air supply. After several hours of adaptation, the caps were replaced with blue NM. After 24 h, caps containing blue NM were replaced with clean, empty ones. Flies were homogenized with 500 µl of water using a bead ruptor (OMNI International, Kennesaw, GA, USA). The homogenate was centrifuged at 3000 x *g* for three minutes to pellet the tissue debris and then transferred into 96-well plates. The absorbance at 630 nm was quantified using a SYNERGY H1 microplate reader (BioTek Instruments, Winooski, VT, USA). A standard curve created via a dilution series of the blue food was used to calculate the amount of ingested food.

### Measurement of fecal output

Standard *Drosophila* vials were placed tilted and filled with ~ 3 ml of blue-dyed NM (0.5% [w/v] Brilliant Blue FCF) and covered with a cover slip (24 × 50 mm). Three female flies were placed in each experimental vial. The cover slip was fixed with a plug that served as the bottom. After 24 h, all fecal spots were counted and the number of fecal spots per fly was calculated.

### Body composition

The body fat of flies was measured using the coupled colorimetric assay from [31] as described by [32]. The protein content was determined by using a Pierce BCA Protein Assay Kit (Thermo Fisher Scientific, Karlsruhe, Germany) according to the manufacturer’s protocol.

### Activity monitoring

Fly activity was measured using the *Drosophila* Activity Monitor System (DAM; TriKinetics. Waltham, MA, USA) as previously described [33] with minor modifications. Individual flies were transferred into glass tubes filled with NM. Tubes were placed horizontally into the DAM device. After adapting to the conditions for one day, their activity was monitored for three days. The data was analyzed using the web application ShinyR-DAM [34].

### Metabolic rate

The metabolic rate of individual adult female flies was determined by direct microcalorimetry in a TAM IV instrument equipped with six 4 ml microcalorimeters (TA Instruments) as previously described [35]. In detail, heterozygous *dSirt4* mutant flies were backcrossed to *w*^*1118*^ (BDSC; #5905) flies for three generations before *w*^*1118*^*TI{w*^*+ mW.hs*^*=TI}Sirt4*^*white + 1*^*/ w*^*1118*^ and *w*^*1118*^ virgin females were crossed to *w*^*1118*^*TI{w*^*+ mW.hs*^*=TI}Sirt4*^*white + 1*^*/ Y* and *w*^*1118*^ males, respectively. The fertilized females were pooled in the same NM vial, and then homozygous *Sirt4* mutant and *w*^*1118*^ control F1 females were mated. Afterward, they were kept separately from the males at 25 °C in constant darkness and at 75% relative humidity for six days on NM. Individual flies were transferred to disposable 4 ml crimp seal glass ampoules that were preloaded with 200 µl NM. The heat dissipation of six flies per genotype (two runs of three mutants and three controls on two consecutive days) was measured over four hours. After the microcalorimeter run, the wet weight of the flies was determined using a Sartorius MC5 balance. The heat dissipation of individual flies was averaged per hour, and the metabolic rate was calculated in mJ/h/mg wet weight.

### Sample preparation for proteomic analysis

*Drosophila* intestines of *dSirt4* knockout flies and control flies (*w*^*1118*^) were analyzed by bottom-up proteomics and label-free quantification. *Drosophila* intestines were prepared as described previously [36]. Cell disruption was performed in 5 µl of lysis buffer (6 M urea, 100 mM tetraethylammonium bromide, and 1× complete protease inhibitor) per *Drosophila* intestine with glass beads. First, samples were processed using a Bioruptor Pico with ten cycles of 30 s of sonication and 30 s of cooling, followed by vortexing for 10 s and freezing at − 80 °C. These disruption steps were repeated five times before the protein concentration was determined by BCA assay. Aliquots of 20 µg protein were reduced for one hour at 60 °C with 20 mM Tris (2-carboxyethyl) phosphine, alkylated for 30 min with 40 mM chloroacetamide, and digested overnight with 0.5 µg of trypsin. After digestion, the samples were acidified with 0.1% trifluoroacetic acid (TFA), lyophilized until dry, redissolved in 100 µl 0.1% TFA, and cleaned using 100 µl C18-tips according to the manufacturer’s protocol (Pierce). Then, the samples were re-lyophilized until dry, dissolved in 20 µl 0.1% TFA, and transferred to vials for separation through high-performance liquid chromatography.

### LC–MS analysis

For the LC–MS analysis, approximately 1 µg of peptides were loaded on a C18 precolumn (PepMap100, 5 μm, 300 Å, Thermo Fisher Scientific) and separated on a C18 column (50 cm × 75 μm, 2.6 μm, 100 Å, Thermo Fisher Scientific) using a Dionex U3000 UHPLC system (Thermo Fisher Scientific) coupled to a Q Exactive HF mass spectrometer (Thermo Fisher Scientific). The separation was performed across a 2.5-hour gradient with eluent A (water, 0.05% formic acid) and eluent B (80% acetonitrile, 0.04% formic acid) with a flow rate of 0.3 µl/min. The steps were as follows: 5 min in 5% B, 80 min to 20% B, 65 min to 50% B, 5 min to 90% B, isocratic at 90% B for 10 min, and equilibration for 10 min at 5% B.

Full MS spectra were acquired with the following settings: resolution = 60,000, mass range = 300–1600 m/z, RF lens = 30%, automatic gain control (AGC) target = 3E6, and maximum injection time = 100 ms. MS2 spectra of the top 10 precursors with a charge state > 2 and < 8 were acquired, with an isolation window of 2 m/z, resolution of 15,000, AGC target of 2E5, injection time of 100 ms, and normalized collision energy of 28. Dynamic exclusion was enabled with an exclusion duration of 10 s.

In total, 21 samples were analyzed, and a database search and label-free quantification were conducted with Proteome Discoverer software (version 3.0.1.27; Thermo Fisher Scientific). The raw data were searched against the combined UniProt protein databases for *Drosophila melanogaster* (UniProt 05.2023; 22,066 entries), the defined microbiota bacteria—*Lactobacillus plantarum* (UniProt 07.2021; 3,179 entries), *Lactobacillus brevis* (UniProt 07.2021; 2,201 entries), *Acetobacter pomorum* (UniProt 07.2021; 2,815 entries), *Commensalibacter intestini* (UniProt 07.2021; 2,209 entries), and *Enterococcus faecalis* (UniProt 07.2021; 3,240 entries)—and common contaminants. The Sequest HT and Chimerys search algorithms were used. The Sequest search parameters were semi-tryptic protease specificity with a maximum of four missed cleavage sites. The precursor mass tolerance was 10 ppm, and the fragment mass tolerance was 0.04 Da. Oxidation of methionine and acetylation of lysine residues and protein N-termini were allowed as dynamic modifications. Carbamidomethylation of cysteine was set as a static modification. The default settings were used for the Chimerys database. Percolator q-values were used to restrict the false discovery rate (FDR) of peptide spectrum matches to 0.01. The FDR of peptide and protein identifications was restricted to 1%, and strict parsimony principles were applied to protein grouping. Label-free quantification was performed with the Minora feature detector. Label-free intensities (LFIs) were based on the precursor intensities of MS spectra, and unique and razor peptides were used to calculate the LFIs of protein groups.

Statistical analyses were performed with the Perseus software. The dataset was filtered for 2,313 protein groups with at least six valid LFIs. The LFIs were log_2_ transformed, and missing values were replaced from a normal distribution (width: 0.3, downshift: 2). The datasets of *dSirt4* knockout and control samples grown on defined microbiota were tested for differentially abundant proteins with and without imputation using Welch’s *t*-test and corrected for multiple testing by permutation-based FDR analysis with 250 randomizations. Proteins with a q-value < 0.05 and a 2-fold or 1.5-fold difference were set as two classifications of differential abundance. Fisher’s exact test was used to analyze the annotation enrichment of GO terms, InterPro classifications and UniProt Keywords among proteins categorized as higher and lower abundant in *Sirt4* KO mutants. The annotation enrichment analyses were corrected for multiple testing using Benjamini-Hochberg FDR calculation.

The results of the proteome analysis and enrichment analyses are provided in the supplementary table S1. MS data were deposited to the ProteomeXchange Consortium [37] by the PRIDE partner repository (dataset identifier: PXD054704 (access for reviewer only: Username: reviewer_pxd054704@ebi.ac.uk Password: O9RH0Xw5oR4O).

### Lysozyme assay

To measure lysozyme activity, homogenates of *Drosophila* intestines were pipetted onto agarose plates containing cell walls of *Micrococcus lysodeikticus.* Plates were prepared by mixing 0.05 M sodium acetate (NaAc) with 0.9% agarose and boiling. Next, 0.6 mg/ml *M. lysodeikticus* (American Type Culture Collection No. 4698; M3770-5 g, Sigma-Aldrich) was dissolved in 1 ml NaAc at 37 °C with shaking. After the agarose had cooled to under 50 °C, the *M. lysodeikticus* solution was added, and the mixture was poured into petri dishes. Five intestines were dissected and homogenized in 50 µl phosphate-buffered saline (PBS) using a bead ruptor (Bead Ruptor 24, OMNI International). Then, the homogenate was added into holes punched into the plates. The diameter of the lysis zone was measured after 24 h of incubation at 37 °C.

### Dechorionization and recolonization

For egg deposition, flies were placed on apple juice agar and several chunks of fresh yeast mixed with a few drops of apple vinegar. After 18 h at 20 °C, eggs were dechorionated with 6% sodium hypochlorite for five minutes, sprayed with 70% ethanol, rinsed with sterile water, and placed onto sterile NM without propionic acid. The germfree embryos were recolonized with a mixture of six bacterial species: *L. plantarum*^*WJL*^, *L. brevis*^*EW*^, *A. pomorum*, *C. intestini*^*A9111T*^, *E. faecalis* (all kind gifts from Carlos Ribeiro), and *A. thailandicus* (a gift from Luis Teixeira). The culturing and the adjustment of specific optic densities were performed as described [38]. Each *Drosophila* vial was inoculated with 50 µl of the bacterial suspension.

### Microbial community analysis

After the recolonized flies hatched, they were inoculated with 50 µl of the bacteria mixture and transferred to sterile NM every 3–4 days. On day 10, six replicates with 5–6 intestines per group were dissected. Genomic DNA was extracted using the DNeasy Blood and Tissue Kit (Qiagen) following the protocols “Pretreatment for Gram-Positive Bacteria” and “Purification of Total DNA from Animal Tissues”. The extracted DNA was eluted in 50 µL of AE buffer. Bacteria-specific primers were used to check for the presence of bacterial DNA (V2-F: AGAGTTTGATCCTGGCTCAG, V2-R: TGCTGCCTCCGGTAGGAGT).

The V1–V2 region of the 16 S rRNA gene was amplified following the guidelines described by Rausch et al. [39, 40] and subjected to 250 bp paired-end sequencing on the Illumina MiSeq platform. Each sample’s sequences were allocated based on precise matches to multiplex identifier sequences and processed using the *dada2* package (v.1.32.0) [41] in R statistical software. Briefly, two “expected errors” were permitted in a read after raw sequences were cut and filtered for quality. After merging the paired reads, chimeras were removed before taxonomy was assigned, including species-level assignments, using the Silva training set (nr 99 v138). Classifications with low confidence at the genus level (< 0.8) were grouped under the arbitrary taxon “unclassified\_group.” Contaminants were removed using the prevalence and frequency functions of the *decontam* R package (v.1.24.0) [42]. Bacterial load qRT-PCR measurements were used as a proxy for DNA quantity for the frequency function. Samples were rarified to 36,400 reads. Alpha (Shannon) and beta (Bray–Curtis) diversity were analyzed using the *phyloseq* R package (v.1.48.0) [43]. A pairwise Wilcoxon test was used to test for differences in alpha diversity between groups, and a permutational multivariate analysis of variance with 10,000 permutations was used to test for differences in beta diversity. Differentially abundant genera between respective groups and WT were tested using the *limma voom* method from the R package *microbiomeMarker* (v 1.13.2). P-values were adjusted using the FDR adjustment method.

### Bacterial load assay

Flies were dechorionated and recolonized as described above. For the association with only two bacterial strains, embryos were recolonized with 50 µl of *A. thailandicus* and *L. plantarum* cultures (OD_600_ = 2). After hatching, flies were inoculated with fresh bacteria solution and transferred to sterile NM every 3–4 days. On day 10, groups of three flies were homogenized in 100 µl of sterile PBS. Five dilutions (up to 1:10,000) were plated on MRS, LB, and mannitol agar plates [38].

### Total RNA extraction

Total RNA was extracted using RNAmagic and the Ambion PureLink RNA Mini Kit (Thermo Fisher Scientific). Eight to ten dissected *Drosophila* intestines were homogenized in 1 ml of RNAmagic (Bio-Budget Technologies GmbH, Krefeld, Germany) using a bead ruptor (OMNI International). After incubation at room temperature for five minutes, 200 µl of chloroform was added. Samples were shaken for 10 s, incubated on ice for five minutes, and centrifuged at 4 °C and 12,000 x *g* for 15 min for phase separation. Next, 400 µl of the upper phase containing the RNA was transferred into a 1.5 ml reaction tube. The RNA was purified according to the manufacturer’s protocol, “Purifying RNA from Animal Tissue: Binding, Washing, and Elution steps.” Samples were eluted in 30 µl of RNase-free water and stored at − 80 °C. The mRNA was reverse transcribed to generate cDNA using SuperScript IV Reverse Transcriptase (Thermo Fisher Scientific).

### qRT-PCR

qRT-PCR was performed using the qPCRBIO SyGreen Mix Hi-Rox (PCR Biosystems, London, UK), MicroAmp Optical 96-well Reaction plates (0.1 ml, Thermo Fisher Scientific), and the QuantStudio 1 Real-Time PCR System (Thermo Fisher Scientific). The following primers were used: *dSirt2* (GGATTTCAGATCCCCAGGTT and GATCGAATATGGCCGTAGGA), *dSirt4* (CCGAAATGTTGTGGAGGTTC and ATTTAGCGACGCCAGTATGC), *dSirt6* (TGGATTGTCAGCCTACGACA and GACAACGTGTCCCGATTTCT), *dSirt7* (GAGGAAACGCAAGACTCGAC and CTGTCGGAGCTCCAGGTTAG), *Drosomycin* (ACCAAGCTCCGTGAGAACCTT and TTGTATCTTCCGGACAGGCAG), *Metchnikowin* (CCACCGAGCTAAGATGCAA and AATAAATTGGACCCGGTCTTG), *Diptericin* (GCAATCGCTTCTACTTTGGC and TAGGTGCTTCCCACTTTCCA), *Attacin-A* (TTCCGTGAGATCCAAAG and CAATCTGGATGCCAAGGTCT), *Cecropin* (AAGATCTTCGTTTTCGTCGC and GTTGCGCAATTCCCAGTC), *Drosocin* (GTTCACCATCGTTTTCCTGC and GGCAGCTTGAGTCAGGTGAT), *Def* (GCTATCGCTTTTGCTCTGCT and GCCGCCTTTGAACCCCTTGG), *Lysozym P* (CCAGGCCCGAACGATGGATAGGT and CGGGGAACGCCCAGTTTGGA), and ribosomal protein L32 (*RpL32*; CCGCTTCAAGGGACAGTATC and GACAATCTCCTTGCGCTTCT).

### Statistical analysis

The lifespan data was analyzed for statistical significance using the log-rank (Mantel–Cox) test. All other data were first assessed for a normal Gaussian distribution using the Shapiro–Wilk test and then compared using an unpaired *t*-test (normally distributed data) or Mann–Whitney test (non-normally distributed data).

## Results

**Fig. 1:**
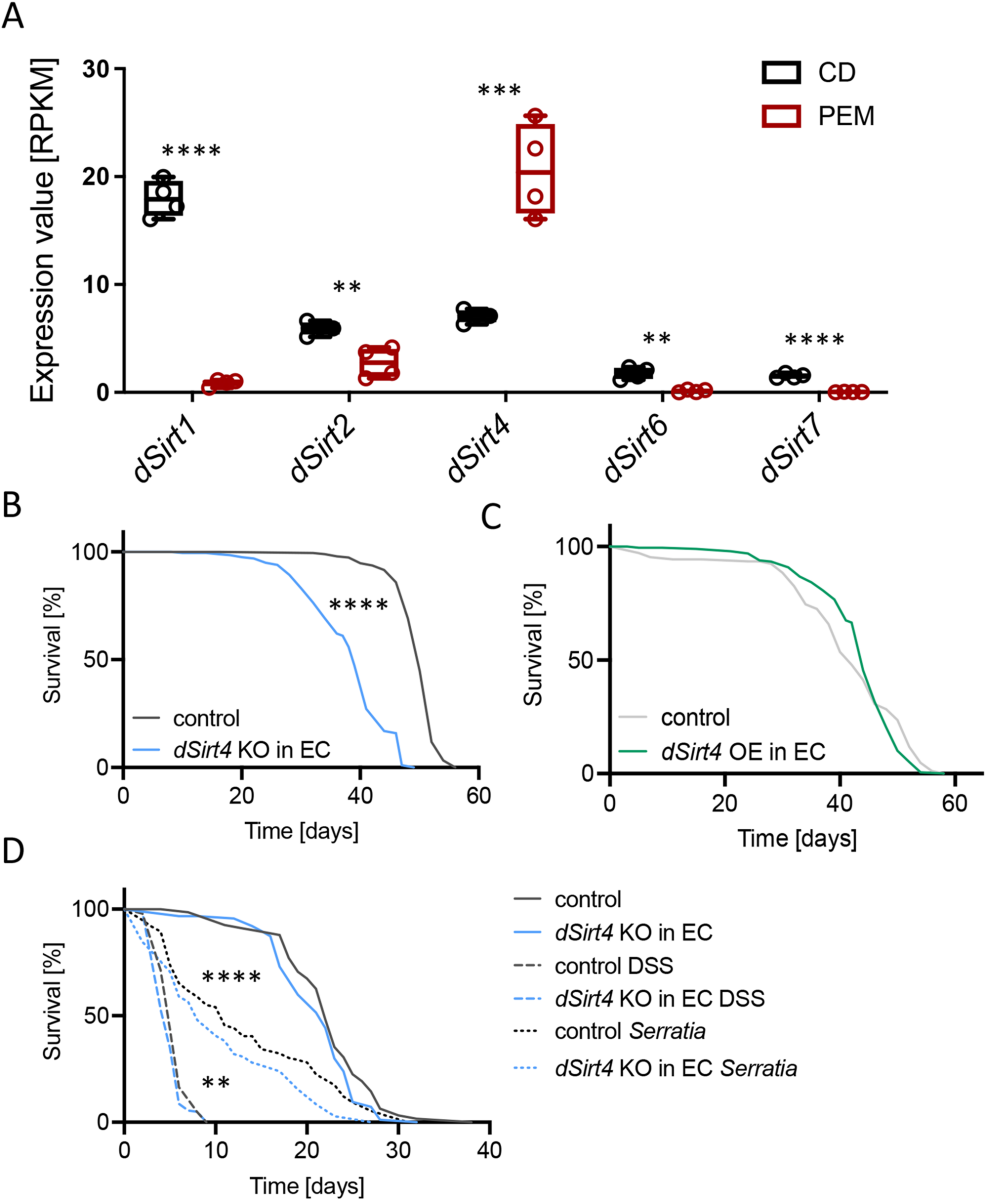
dSirt4 has an important role in the stress response of the *Drosophila* intestine. (**A**) A gene expression analysis of isolated intestines of flies subjected to 7 days protein depleted diet (PEM) revealed that the only sirtuin gene upregulated under these conditions is the only mitochondrial sirtuin of *Drosophila*, *dSirt4*, while all others were downregulated (CD = control diet, *n* = 4). (**B**) A lifespan analysis showed a significantly reduced survival of flies with a knockout of *dSirt4* specifically in enterocytes (*dSirt4* KO in EC, *n* = 187–195). (**C**) The overexpression of *dSirt4* in enterocytes did not affect the lifespan of flies (*dSirt4* OE in EC, *n* = 91–185). (**D**) *dSirt4* knockout flies showed reduced survival in response to infection with *Serratia marcescens*, while the control treatment with sucrose did not lead to changes in lifespan (*n* = 63–104). In response to the treatment with DSS, *dSirt4* knockout flies lived shorter than their genetic control (*n* = 63–186). (**B**, **D**) control = *w*^*1118*^ *> UAS-sirt4 crispr/Cas9*, *dSirt4* KO in EC = *NP1-Gal4;tubPGal80ts > UAS-sirt4 crispr/Cas9*, (**C**) control = *w*^*1118*^ *> UAS-sirt4*, *dSirt4 OE* in EC = *NP1-Gal4;tubPGal80ts > UAS-sirt4.* * = *p* < 0.05, ** = *p* < 0.01, **** = *p* < 0.0001

We conducted a transcriptome analysis to identify particularly interesting sirtuins that adapt to changing dietary conditions in the intestine. We exposed adult female flies (*w*^*1118*^) to different nutritional conditions, specifically substantial food stress. We utilized a holidic diet, described recently [29, 44], which enabled us to maintain all dietary components constant, except for protein content, which was reduced to zero for this experiment. This feeding intervention lasted seven days, after which we isolated the guts of the flies and compared them to age-matched controls. Of the five sirtuins identified in *Drosophila*, only *dSirt4* encodes a mitochondrial sirtuin. It was the only sirtuin significantly upregulated after feeding stress (*p* = 0.0008, unpaired *t*-test), while all other sirtuins were downregulated: *dSirt1* (*p* < 0.0001), *dSirt2* (*p* = 0.0059), *dSirt6* (*p* = 0.0011), and *dSirt7* (*p* < 0.0001; unpaired *t*-test; Fig. 1A). We also evaluated two other stressors: starvation and DSS (Dextran Sulphate Sodium) treatment. In this case, all sirtuins exhibited increased expression in response to DSS treatment (*dSirt1* (*p* < 0.0113; unpaired *t*-test), *dSirt2* (*p* = 0.0079), *dSirt6* (*p* = 0.0079), and *dSirt7* (*p* = 0.0079); Mann-Whitney test; Fig. S1A) and reduced or unchanged expression in response to starvation (Fig. S1B). In response to starvation, *dSirt1* (*p* < 0.0001; unpaired *t*-test) and *dSirt2* (*p* = 0.0079, Mann-Whitney test) showed reduced expression, while all other Sirtuin genes remained unchanged. Based on these results, all subsequent experiments were done with *dSirt4* as it showed the unique property of being upregulated by a relevant stressor.

To directly link *dSirt4* KOs to gut properties, we used CRISPR/Cas9-mediated, cell-specific KOs in combination with genomic KOs. This experimental approach allows us to distinguish between direct effects, which occur in the primary absorptive cells of the intestine, and indirect effects, where *dSirt4* deficiency affects the microbiota in other organs. To characterize the role of *dSirt4* in the intestine, we first measured the lifespan of flies with clustered regularly interspaced short palindromic repeats (CRISPR)/CRISPR-associated protein 9 (Cas9)-mediated knockout of *dSirt4* (specifically in enterocytes). We found that these flies showed significantly reduced survival compared to the upstream activating sequence (UAS)-control flies (median lifespan: 39 vs. 50 days; *p* < 0.0001, log-rank [Mantel–Cox] test; Fig. 1B). However, overexpressing *dSirt4* in the enterocytes did not significantly affect the survival of flies (median lifespan: 42–44 days; Fig. 1C).

Next, we tested whether knocking out *dSirt4* in enterocytes affects the susceptibility of flies to orally administered stressors. These experiments involved infection with *Serratia marcescens*, an entomopathogenic gram-negative bacterium [36]. Flies with an enterocyte-specific knockout of *dSirt4* exhibited significantly shorter lifespans than the UAS-control flies under constant bacterial infection (median lifespan: 8 vs. 11 days; *p* < 0.0001, log-rank [Mantel–Cox] test; Fig. 1D). In the infection-free control group, which received only 5% sucrose, the survival rate of *dSirt4* knockout flies did not differ significantly from that of the genetic control group (median lifespan = 22 days; Fig. 1D). We explicitly used only the UAS control to allow for a straightforward statistical analysis.

Dextran sodium salt (DSS) is a substance used to induce colitis in mice [45]. DSS also causes tissue damage and proliferation in *Drosophila* and reduces their lifespan [46]. As expected, flies fed a 5% sucrose solution containing 5% DSS showed significantly reduced survival compared to the DSS-free control flies. This reduction was slightly, but significantly, more pronounced in flies with *dSirt4* knocked out (*p* = 0.0072, log-rank [Mantel–Cox] test; Fig. 1D). Compared to the genetic control flies, the median survival of *dSirt4* knockout flies was reduced from 6 to 5 days.

**Fig. 2:**
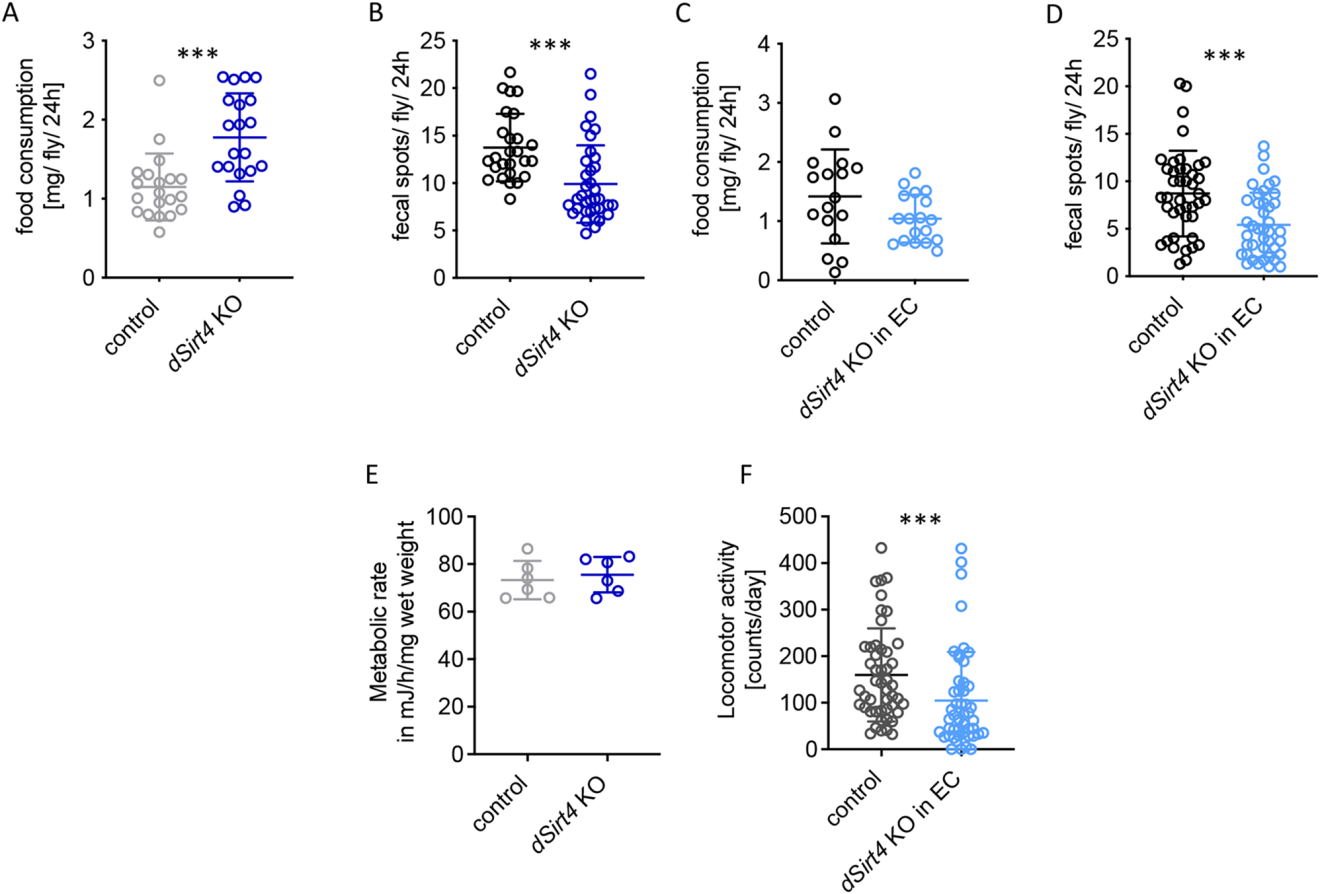
Changes in gut functionality upon reduced *dSirt4* expression. (**A**, **B**) The knockout of *dSirt4* (*dSirt4* KO) leads to an increase in food consumption (*n* = 20), while the number of excreted fecal spots is not affected (*n* = 26–36). (**C**, **D**) In flies with a knockout of *dSirt4* in enterocytes (*dSirt4* KO in EC), the amount of ingested food is not significantly different compared to the control (*n* = 17–18), but the number of excreted fecal spots is reduced (*n* = 41). (**E**) The metabolic rate of *dSirt4* KO flies was determined by measuring the heat dissipation and showed no significant differences to controls (*n* = 6). (**F**) Flies with a knockout of *dSirt4* in enterocytes have a significantly reduced locomotor activity (*n* = 46–48). (**A**, **B**, **E**) control = *w*^*1118*^. (**C**, **D**, **F**) control = *w*^*1118*^ *> UAS-sirt4 crispr/Cas9*, *dSirt4* KD = *NP1-Gal4;tubPGal80ts > UAS-sirt4 crispr/Cas9.* *** = *p* < 0.001

To further characterize the role of *dSirt4*, we analyzed gut functionality by quantifying daily food consumption [30]. *dSirt4* knockout flies consumed significantly more food than the control flies (*p* = 0.0001, Mann–Whitney test; Fig. 2A), but they excreted significantly fewer fecal spots (*p* < 0.0001, Mann–Whitney test; Fig. 2B). However, we did not collect information on the shape and volume of individual fecal spots. The amount of ingested food did not differ significantly between flies with enterocyte-specific knockout of *dSirt4* and the UAS-control flies (Fig. 2C). However, they excreted significantly fewer fecal spots (*p* = 0.0005, Mann–Whitney test; Fig. 2D). The metabolic rate was evaluated by measuring heat dissipation, which did not differ significantly between *dSirt4* knockout flies and the control flies (Fig. 2E). Finally, we measured the spontaneous locomotor activity of flies. Flies with enterocyte-specific knockout of *dSirt4* showed significantly reduced activity compared to control flies (*p* = 0.0005, Mann–Whitney test; Fig. 2F).

**Fig. 3:**
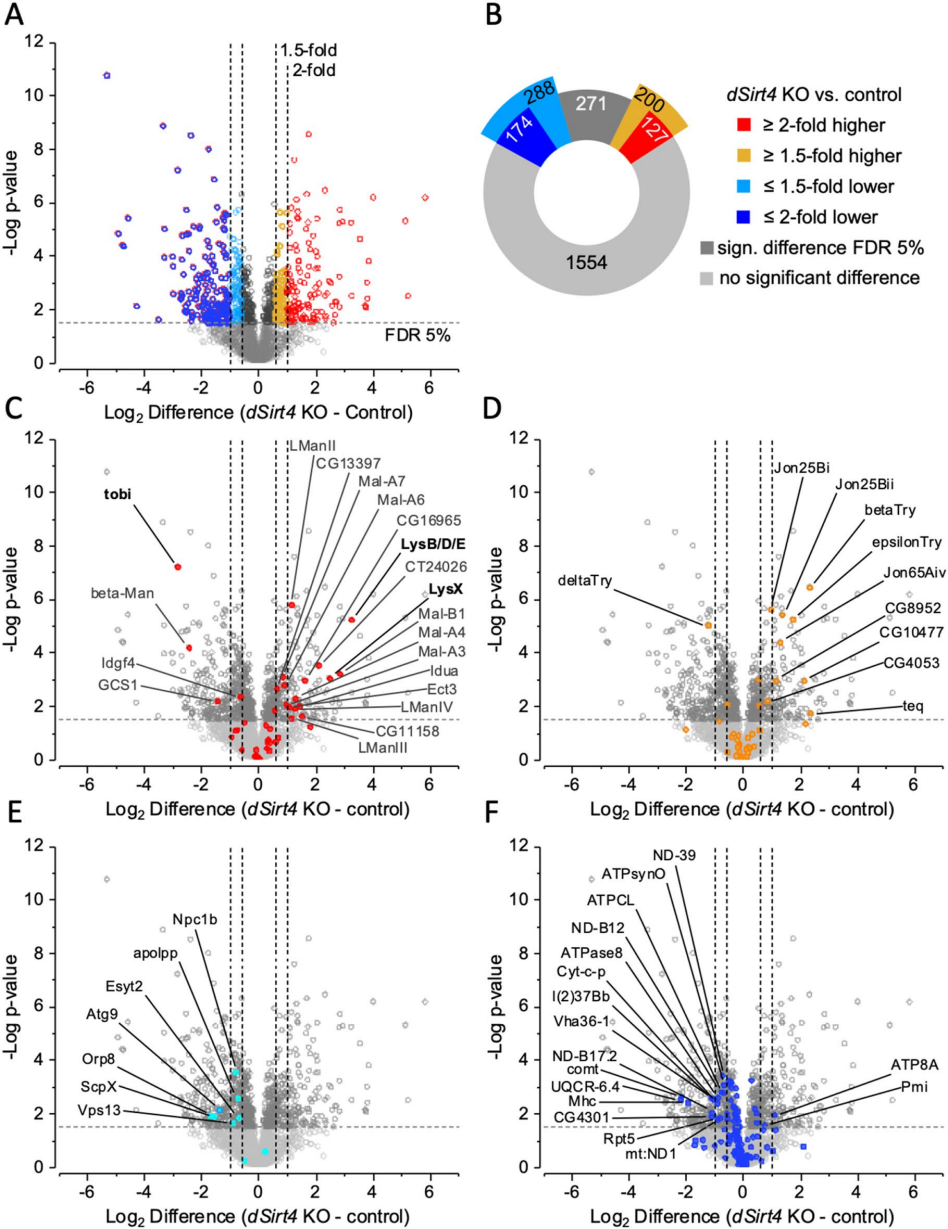
Quantitative proteome analysis of adult intestines. (**A**, **B**) Differentially abundant proteins were detected by label-free quantification in *dSirt4* KO intestines (*n* = 6) compared to the control strain (*n* = 7) of *Drosophila*. (C-F) Show the distribution of proteins associated with distinct functional categories labelled by colors; significantly changed proteins are labelled by their naming. (**C**) Including the protein group encompassing LysB, LysD and LysE, glycosidases were significantly more abundant, whereas the carbohydrate regulatory protein Tobi was less abundant in *dSirt4* KO. (**D**) Digestive serine proteases were more abundant in *dSirt4* KO intestines. (**E**) Most proteins involved in lipid transport were of significantly lower abundance in the *dSirt4* KO. (**F**) Proteins associated with mitochondrial electron transport, the respiratory chain complex, and ATP synthases showed a trend towards lower abundance in *dSirt4* KO intestines. control = *w*^*1118*^

The effect of *dSirt4* knockout on the intestinal proteome of *Drosophila* was analyzed with label-free quantitative proteomics using liquid chromatography–mass spectrometry (LC–MS). In 21 analyzed samples, a total of 2,881 protein groups were identified, of which 2,364 could be quantified. Between the *dSirt4* knockout flies and control flies, 301 proteins showed a greater than two-fold difference in abundance, and 488 showed a greater than 1.5-fold difference (Figs. 3A and B). The complete dataset is provided in Supplementary Table S1. After adjusting p-values for multiple testing, no GO terms or pathways were significantly enriched in any subset of differentially abundant proteins. However, a few UniProt Keywords and InterPro protein families were enriched. The results of enrichment analyses are also shown in the Supplementary Table S1.

The most notable result was the increased abundance of members of the lysozyme family, which play a role in digestion and microbial control [47]. Due to appreciable sequence similarities, lysozymes B *(LysB*), D (*LysD*), and E (*LysE*) could only be detected as one protein group, which was much more abundant (9.5-fold) in the intestines of *dSirt4* knockout flies compared to control flies. In addition, lysozyme X (*LysX*) was found at significantly higher abundances in *dSirt4* knockout flies (Fig. 3C). Glycosidases were significantly enriched, with 16 members among the 200 proteins showing at least a 1.5-fold higher abundance in *dSirt4* knockout flies. These included several lysosomal glycosidases for various substrates (Fig. 3C). The same trend was observed in proteases. Ten serine proteases (Fig. 3D) and four metalloproteases were more abundant in the *dSirt4* knockout flies. In contrast, *dSirt4* knockout flies had lower abundances of almost all of the proteins involved in lipid transport (Fig. 3E). These data show that even without a change in diet, knocking out *dSirt4* significantly alters the metabolic state of the fly. Interestingly, proteins annotated to the mitochondrial electron transport, the respiratory chain complex, and ATP synthases showed a noticeable trend toward lower abundance in *dSirt4* knockout flies than in control flies (Fig. 3F).

**Fig. 4:**
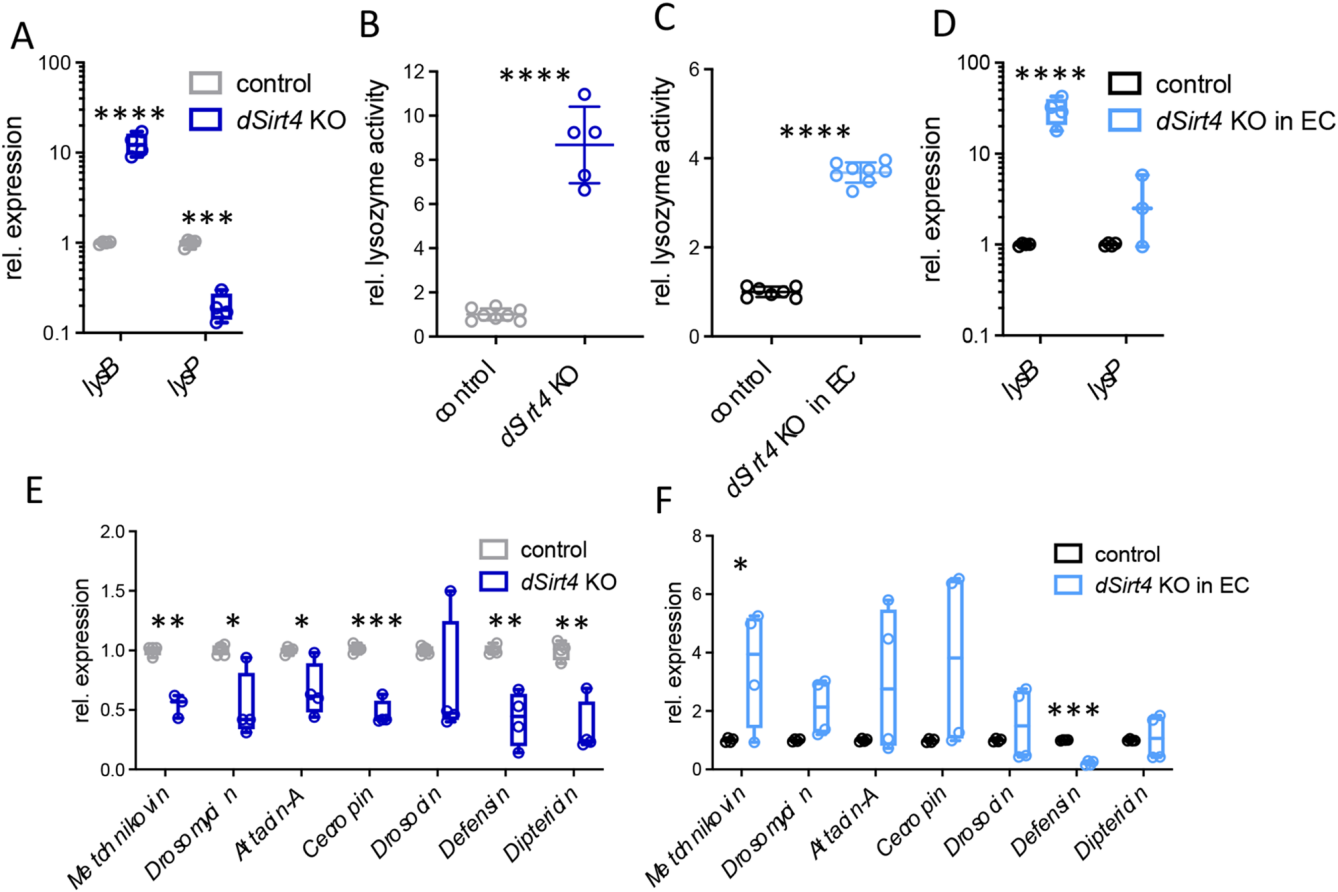
Deficiency of *dSirt4* strongly induces lysozyme expression in the intestine. (**A**) qRT-PCR analysis showed significantly upregulated *lysB* expression in *dSirt4* KO intestines (*n* = 4). (**B**) Strong increase of lysozyme activity in *dSirt4* KO intestines (*n* = 5–8). (**C**) Increase of lysozyme activity (*n* = 8) and (**D**) upregulation of *lysB* expression in intestines of flies with a *dSirt4* knockout in enterocytes (*n* = 4). (**E**, **F**) Changes in expression of antimicrobial peptide genes in intestines from *dSirt4* KO flies (**E**; *n* = 4) and intestines of flies with a *dSirt4* knockout in enterocytes (**F**; *n* = 4). (**A**, **B**, **E**) control = *w*^*1118*^. (**C**, **D**, **F**) control = *w*^*1118*^ *> UAS-sirt4 crispr/Cas9*, *dSirt4* KO in EC = *NP1-Gal4;tubPGal80ts > UAS-sirt4 crispr/Cas9.* * = *p* < 0.05, ** = *p* < 0.01, ***= *p* < 0.001, **** = *p* < 0.0001

To confirm the increased abundance of lysozymes observed in the proteomics analysis, we performed a quantitative real-time reverse transcription-polymerase chain reaction (qRT-PCR) analysis to measure *lysB* and *lysP* expression. As expected, *lysB* expression was significantly upregulated (*p* = 0.0007, unpaired *t*-test), whereas *lysP* expression was downregulated considerably (*p* < 0.0001, unpaired *t*-test; Fig. 4A). Next, we measured the lysozyme activity in dissected intestines by applying the homogenate to petri dishes containing agarose mixed with cells walls from *Micrococcus lysodeikticus* and quantifying the zone of lysis. Intestines from *dSirt4* knockout flies showed significantly higher lytic activity (> 800%) than the control flies (*p* < 0.0001, unpaired *t*-test; Fig. 4B), which is consistent with the difference in protein abundance detected by proteomics. We also investigated whether flies with enterocyte-specific knockout of *dSirt4* had more significant lysozyme activity. The zone of lysis was also significantly larger for these flies (~ 350%), but not to the extent of the *dSirt4* knockout flies (*p* < 0.0001, unpaired *t*-test; Fig. 4C). A qRT-PCR analysis further revealed an upregulation of *lysB* expression (*p* = 0.0012, unpaired *t*-test; Fig. 4D).

We measured the expression of different antimicrobial peptide genes in the intestine using qRT-PCR, to test if the increased lysozyme activity is part of a broader immune system activation. The expression of *Metchnikovin*, *Drosomycin*, *Defensin*, *Diptericin*, *Attacin-A*, and *Cecropin* was significantly downregulated in *dSirt4* knockout flies compared to control flies (*p* = 0.0086, 0.0159, 0.0259, 0.0286, 0.0025, and 0.0286, respectively; Mann–Whitney test or unpaired *t*-test), only the expression of *Drosocin* was not significantly affected (Fig. 4E). In flies with enterocyte-specific knockout of *dSirt4*, only the expression of *Metchinokovin* was upregulated considerably (*p* = 0.0474, unpaired *t*-test), while the expression of *Defensin* was downregulated (*p* < 0.0001, unpaired *t*-test; Fig. 4F); the expression of the other genes did not change significantly.

To test whether the effect of Sirt4 depletion on lysozyme expression and activity is unique to *dSirt4* or if it is also observed for other sirtuins, we also analyzed the impact of knocking out the two other sirtuin genes (*dSirt1* and *dSirt2*) that are substantially expressed in the intestine. This experiment showed that deletions of the *dSirt1* (*p* < 0.0001) and the *dSirt2* gene (*p* < 0.0001; unpaired *t*-test) resulted in a corresponding increase in lysozyme activity (Fig. S1C).

**Fig. 5:**
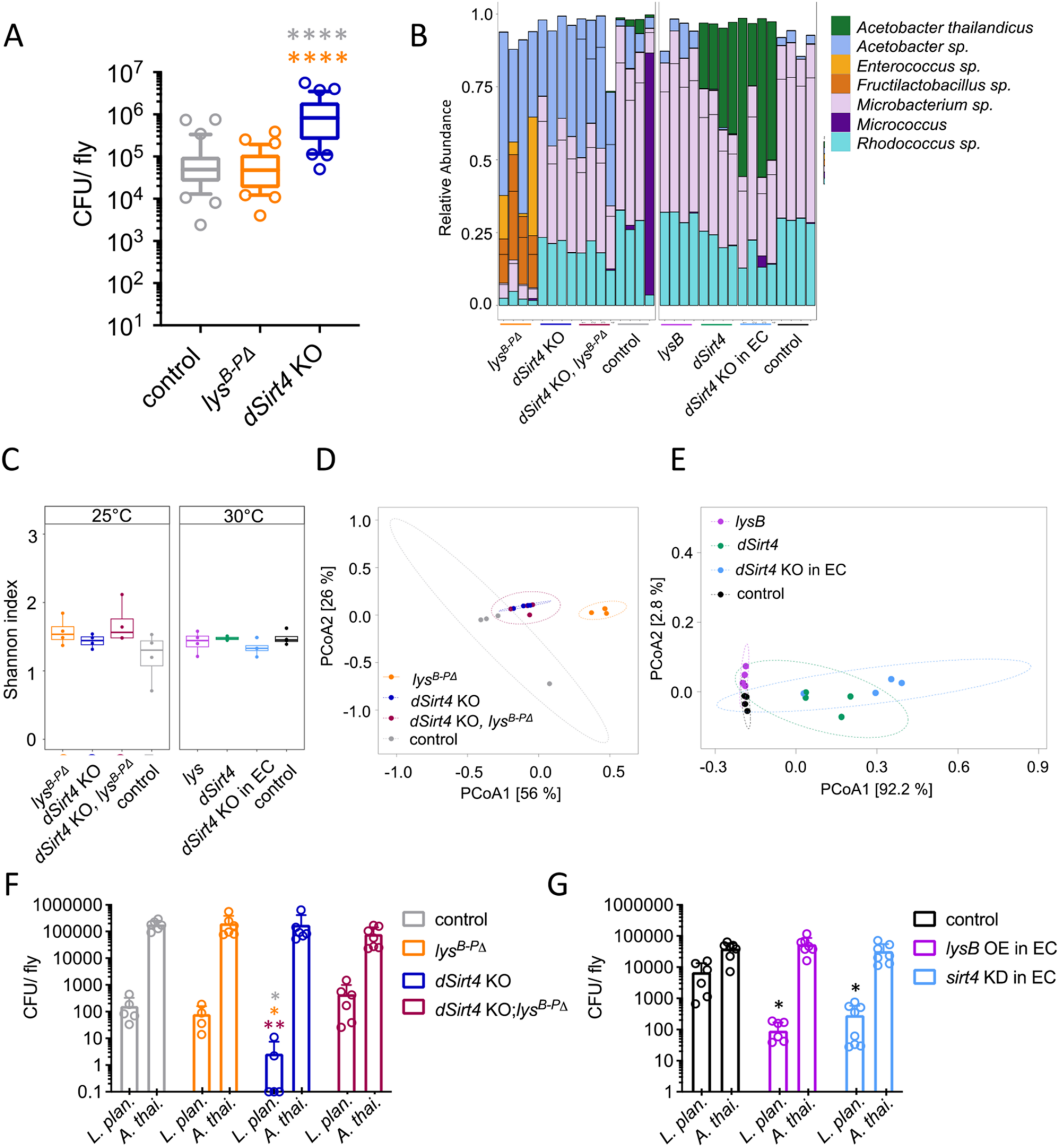
Changes in the bacterial load and composition of the intestinal microbiome upon *dSirt4* and lysozyme manipulation. (**A**) Bacterial load of recolonized *dSirt4* KO, *lys*^*B-PΔ*^, and control flies (*n* = 33). (**B**) Top 10 ASVs, colored according to bacterial species, black lines represent different ASVs belonging to the same species, (**C**) Box plot of the Shannon index and (**D**, **E**) PCoA of the Bray-Curtis dissimilarity of *dSirt4* KO, *lys*^*B-PΔ*^, *dSirt4* KO; *lys*^*B-PΔ*^, *lysB*, *dSirt4* and *dSirt4* KD and control (*n* = 4). (**F**) Reduced number of CFU of *L. plantarum* and *A. thailandicus* after recolonization in disassociation *dSirt4* KO flies compared to control, *lys*^*B-PΔ*^ flies and *dSirt4* KO; *lys*^*B-PΔ*^ flies (*n* = 6). (**G**) Reduced number of CFU of *L. plantarum* and *A. thailandicus* after recolonization in disassociation of flies with *dSirt4* deficiency or overexpression of *lysB* in EC compared to control (*n* = 6). (**A**) control = *w*^*1118*^. (**B**-**E**) control (grey) = *w*^*1118*^, control (black) = *NP1-Gal4;tubPGal80ts > w*^*1118*^, *lysB = NP1-Gal4;tubPGal80ts > UAS-lysB*,* dSirt4 = NP1-Gal4;tubPGal80ts > UAS-sirt4*,* dSirt4* KD = *NP1-Gal4;tubPGal80ts > UAS-sirt4 crispr/Cas9.* (**F**) control = *w*^*1118*^. (**G**) control = *NP1-Gal4;tubPGal80ts > w*^*1118*^, *lysB = NP1-Gal4;tubPGal80ts > UAS-lysB*,* dSirt4* KD = *NP1-Gal4;tubPGal80ts > UAS-sirt4 crispr/Cas9.* * = *p* < 0.05, ** = *p* < 0.01, **** = *p* < 0.0001

To test whether the substantial increase in lysozyme activity impacts the intestinal microbiota, we performed bacterial load assays with *dSirt4* knockout, lysozyme-deficient (*lys*^*B-P*Δ^), and control flies. Axenic embryos were recolonized with a mix of six *Drosophila* gut bacteria (*Lactobacillus brevis*,* Lactobacillus plantarum*,* Acetobacter pomorum*,* Acetobacter thailandicus*,* Enterococcus faecalis*, *and Commensalibacter intestini*) [38, 48]. After hatching, flies were kept together in one container for five days to ensure the same starting condition. Afterward, flies were sorted and kept for an additional ten days. Homogenates of whole flies were plated onto De Man–Rogosa–Sharpe (MRS)-, Luria–Bertani (LB)-, and mannitol plates, and the number of colony-forming units (CFUs) was determined. Unlike the flies lacking the lysozymes B-P genomic region, the bacterial load was significantly greater for *dSirt4* knockout flies than for control flies (*p* < 0.0001, Mann–Whitney test; Fig. 5A).

Next, we evaluated whether *dSirt4* knockout leads to changes in flies’ intestinal microbial composition. The intestines of recolonized flies were dissected 10 days after hatching, and genomic DNA was extracted and amplified with specific primers for bacterial variable regions 1 and 2 of the 16 S rRNA genes. The amplified DNA was sequenced using an Illumina MiSeq sequencer. The top 10 amplicon sequence variants (ASVs) in the samples differed between *dSirt4* knockout and *lys*^*B-P*Δ^ flies (Fig. 5B) but not between *dSirt4* knockout and double knockout flies. Alpha diversity (Shannon index) did not differ significantly between groups (Fig. 5C). However, the principal coordinates analysis (PCoA) of Bray–Curtis dissimilarity indicated significant differences in beta diversity (Fig. 5D). Differential abundance analysis revealed that *Acetobacter* was significantly more abundant in dSirt4 knockout, *lys*^*B-P*Δ^ flies, and double knockout flies (*p* = 1.912865e-03, *p* = 4.681749e-04, *p* = 8.212204e-04, resp., FDR adjusted, Suppl. Fig X). Additionally, *lys*^*B-P*Δ^ flies had a significantly higher abundance of *Enterococcus* (*p* = 3.004580e-11, FDR adjusted) and *Fructolactobacillus* (*p* = 3.956974e-06, FDR adjusted) and a lower abundance of *Microbacterium (p* = 4.497634e-03, FDR adjusted) and *Rhodococcus* (*p* = 9.822457e-03, FDR adjusted) compared to WT flies. The double knockout showed a higher abundance of *Levilactobacillus* (*p* = 7.070782e-03, FDR adjusted) compared to WT flies. Furthermore, we analyzed the microbiota of flies with enterocyte-specific knockout, overexpression of *dSirt4*, or ectopic overexpression of *lysB*. The top 10 ASVs in flies with enterocyte-specific overexpression or knockout of *dSirt4* differed from those in flies with enterocyte-specific overexpression of *lysB* and the Gal4-control flies (Fig. 5B). The Shannon index indicated no significant differences in alpha diversity between groups (Fig. 5C) but the PCoA of Bray–Curtis dissimilarity indicated significant differences in beta diversity between groups (Fig. 5E). Differential abundance analysis showed a significantly higher abundance of *Levilactobacillus* in *lysB* overexpression flies (*p* = 5.226646e-11, FDR adjusted) and *Acetobacter* in *dSirt4* enterocyte-specific knockout (*p* = 7.489135e-06, FDR adjusted) and overexpression flies (*p* = 8.753804e-05, FDR adjusted, Fig. S3). In addition, *dSirt4* enterocyte-specific knockout had a lower abundance of *Microbacterium* (*p* = 4.329503e-03, FDR adjusted) and *Rhodococcus* (*p* = 1.451138e-03, FDR adjusted) compared to WT flies.

Due to their ability to specifically cleave β-1,4-glycosidic bonds in the peptidoglycan layer, which is thicker in gram-positive bacteria, lysozymes may be involved in altering the ratio of gram-negative and gram-positive bacteria in the microbial community [47]. To test for a shift in the intestinal microbial composition from gram-negative towards gram-positive bacteria caused by the increased activity of lysozymes, we raised flies as described above but recolonized them with a single gram-positive (*L. plantarum*) and a single gram-negative (*A. thailandicus*) bacterial species. The *dSirt4* knockout flies had significantly fewer gram-positive *L. plantarum* bacteria than the control (*p* = 0.0079) and *lys*^*B-PΔ*^ (*p* = 0.0159, Mann–Whitney test) flies (Fig. 5F). The *dSirt4* and *lys*^*B-PΔ*^ double knockout flies also had significantly more gram-positive bacteria than the *dSirt4* knockout flies (*p* = 0.0043, Mann–Whitney test). However, the number of gram-negative *A. thailandicus* did not differ significantly between *dSirt4* knockout and *lys*^*B-P*Δ^ flies compared to control flies, although they were slightly reduced in *dSirt4* and *lys*^*B-PΔ*^ double knockout flies (*p* = 0.020, unpaired *t*-test; Fig. 5F). We also tested flies with enterocyte-specific knockout of *dSirt4* and those overexpressing *lysB*. As expected, both showed significantly fewer gram-positive *L. plantarum* than the Gal4-control flies (*p* = 0.157 and 0.012, respectively; unpaired *t*-test; Fig. 5G). The number of gram-negative *A. thailandicus* did not differ significantly among groups.

**Fig. 6:**
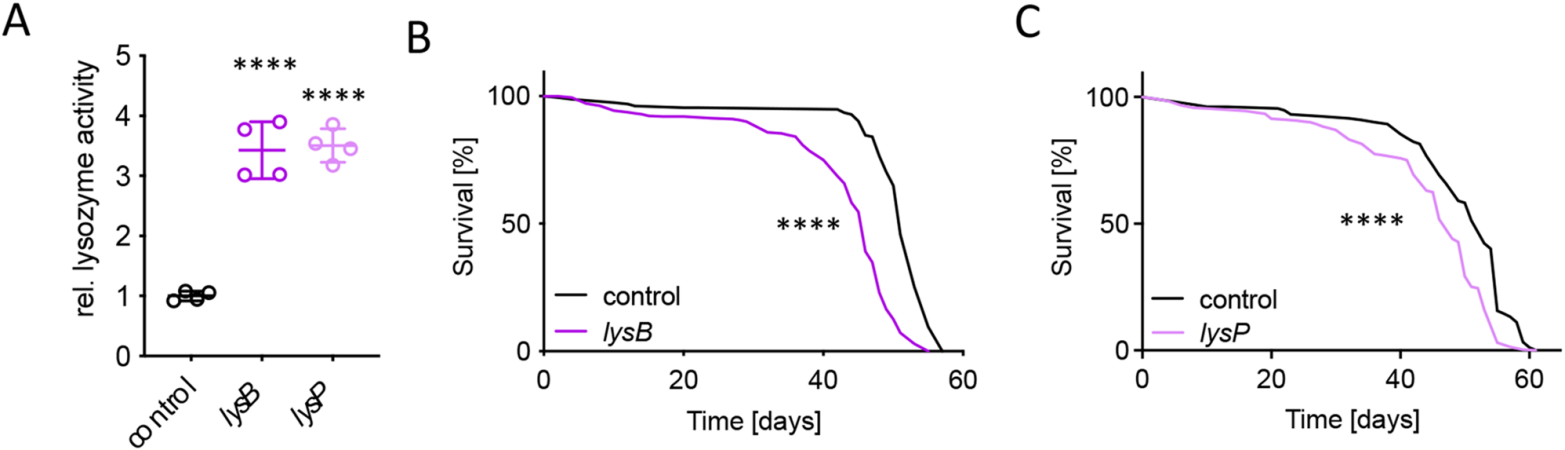
Reduced survival in response to overexpression of lysozymes in enterocytes. (**A**) Increased lysozyme activity in intestines dissected from flies with overexpressed *lysB* or *lysP* in enterocytes (*n* = 4). (**B**, **C**) Reduced survival in flies with an overexpression of *lysB* in enterocytes (**B**; *n* = 150–325) and an overexpression of *lysP* in enterocytes (**C**; *n* = 135–314). control = *NP1-Gal4;tubPGal80ts > w*^*1118*^, *lysB = NP1-Gal4;tubPGal80ts > UAS-lysB*,* lysP = NP1-Gal4;tubPGal80ts > UAS-lysP.* **** = *p* < 0.0001

Since we found that knocking out *dSirt4* in the entire fly and specifically in enterocytes significantly increased lysozyme activity and shortened their lifespan, we examined the impact of increased lysozyme activity on the survival of *Drosophila* by overexpressing *lysB* or *lysP* in the enterocytes. The effectiveness of the overexpression was confirmed using a lysozyme activity assay (*p* < 0.0001, unpaired *t*-test; Fig. 6A). Dissected intestines of flies overexpressing either *lysP* or *lysB* showed markedly increased lysozyme activity (~ 350%). In the survival experiment, flies overexpressing *lysB* in enterocytes had significantly shorter lifespans than the respective UAS-control flies (median lifespan: 46 vs. 51 days; *p* < 0.0001, log-rank [Mantel–Cox] test; Fig. 6B). The overexpression of *lysP* had the same life-shortening effect (median lifespan: 48 vs. 53 days; *p* < 0.0001, log-rank [Mantel–Cox] test; Fig. 6C).

**Fig. 7:**
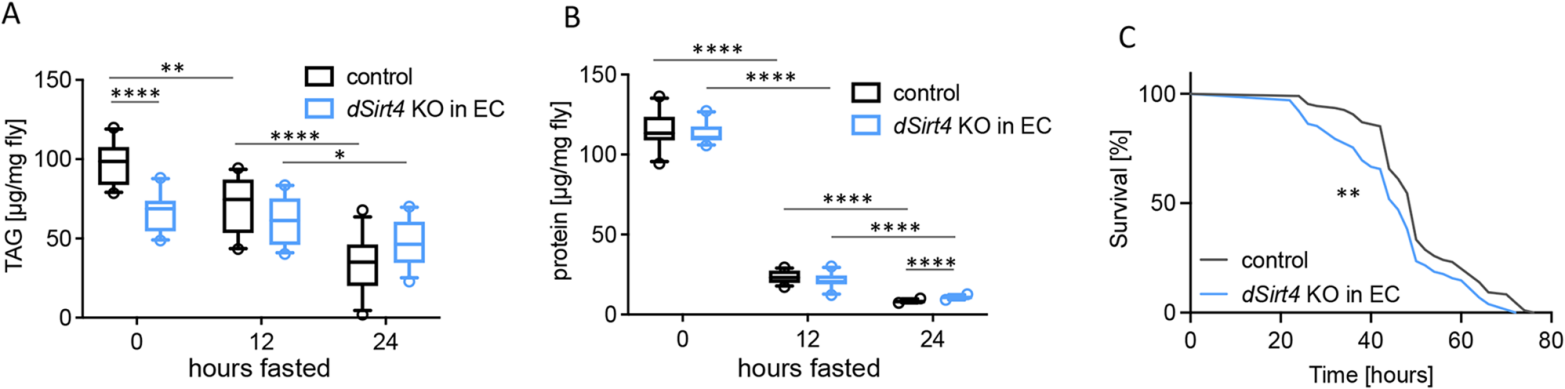
Effects of *dSirt4* on protein and fat metabolism. (**A**) Reduced mobilization of fat storages in response to starvation in flies with a knockout of *dSirt4* in enterocytes (*dSirt4* KO in EC). Body fat content was determined by measuring triacylglycerol (TAG) levels (*n* = 10–12). (**B**) Massive reduction in protein storage upon starvation in *dSirt4* KD flies over a time period of 24 h (*n* = 10–12). (**C**) Reduced survival of flies with a knockdown of *dSirt4* in enterocytes under starvation condition (*n* = 102–108). control = *w*^*1118*^ *> UAS-sirt4 crispr/Cas9*, *dSirt4* KO in EC = *NP1-Gal4;tubPGal80ts > UAS-sirt4 crispr/Cas9.* * = *p* < 0.05, ** = *p* < 0.01, ***= *p* < 0.001, **** = *p* < 0.0001

*dSirt4* knockout flies cannot mobilize stored fat in response to starvation [25]. Therefore, we were interested to see if the enterocyte-specific knockout of *dSirt4* showed similar effects. When we measured the body fat content under control conditions, after 12 h, and after 24 h of starvation, we observed that the triacylglycerol (TAG) content of UAS-control flies decreased significantly in response to starvation (*p* = 0.0011, unpaired *t*-test; Fig. 7A). In contrast, the body fat content of *dSirt4* knockdown flies did not decrease after 12 h of the starvation period, indicating that the flies were unable to mobilize a large part of their fat stores to compensate for the lack of food. The reduction in body fat after 12 and 24 h was 5% and 28% for *dSirt4* knockout flies (*p* = 0.0274, unpaired *t*-test) and 27% and 65% for UAS-control flies (*p* = 0.0011 and *p* < 0.0001, unpaired *t*-test), respectively. We also measured the protein content in response to starvation. The amount of body protein decreased significantly after 12 h (*p* < 0.0001, unpaired *t*-test; Fig. 7B) and even further after 24 h (*p* < 0.0001, unpaired *t*-test). Resistance to starvation, measured by lifespan, was also reduced in flies with enterocyte-specific knockout of *dSirt4* compared to control flies (median lifespan: 46 vs. 50 h; Fig. 7C).

## Discussion

The gut is an organ that forms an interface between the organism and its environment. It directly interacts with one of the most important environmental factors: nutrition. Information on energy content and the main macronutrients is of particular interest [49, 50]. Adapting to conditions and efficiently using the current diet is, therefore, one of the main tasks of the gut [51, 52].

When there is an energy shortage, the gut requires sensors that can bring about significant short- and long-term changes in cell metabolism. Sirtuins, histone deacetylases, can serve as indirect energy sensors through NAD^+^ [9]. Therefore, we focused on identifying highly regulated sirtuins in the intestine during severe nutritional stress, specifically protein malnutrition. Our study revealed that only *dSirt4*, the sole mitochondrial sirtuin in *Drosophila*, responds with a substantial increase in its expression to this nutritional stress, whereas the transcriptomic response to other stressors, such as DSS treatment, is more uniform. We then concentrated on dSirt4 and explored its significance for essential aspects of intestinal biology. Prior research has already demonstrated that dSirt4 significantly impacts lifespan: its overexpression in the fat body extends lifespan, while its knockout shortens lifespan [25].

We demonstrated that *dSirt4* knockout, restricted to the intestinal enterocytes, also shortens lifespan. Our comprehensive proteomic analysis identified several proteins with differential abundances in the intestines of *dSirt4* knockout flies. The massively increased expression of lysozymes was particularly striking. We confirmed this increase at the transcriptome and enzyme activity levels. A similar effect was observed when the *dSirt4* knockout was restricted to enterocytes, showing that the effect is tissue-autonomous. Moreover, the *dSirt4*-dependent increase in lysozyme activity was not part of a complex immune response, as antimicrobial peptide genes were not upregulated under these conditions. However, the mechanism by which decreased *dSirt4* activity increases lysozyme activity remains unclear. Sirtuins can regulate the expression of target genes by different mechanisms, comprising direct modifications of regulatory proteins and the modification of histones, thereby regulating the expression of target genes through altered chromatin accessibility. Which mechanism is operative in the *Drosophila* intestine remains to be elucidated in future studies. Lysozyme regulation by mitochondrial sirtuins appears evolutionarily conserved, as demonstrated by the significant upregulation of *lysP* in response to the gut-specific knockdown of mitochondrial *Sirt3* in mice [15]. Interestingly, the impact of knocking out *dSirt4* on lysozyme expression is not confined to *dSirt4* alone but is also evident in the other two critical intestinal sirtuins, *dSirt1* and *dSirt2*. This observation indicates that the relationship between decreased *dSirt* expression and heightened lysozyme expression is a general phenomenon.

Lysozymes are known for their anti-bacterial activity and are generally classified as anti-bacterial agents due to their ability to lyse gram-positive bacteria by degrading the murein sacculus. The majority of lysozyme expression in the intestine can be assigned to specific enterocyte populations (Fig. S2). Their function, as part of the innate immune response to a range of pathogenic bacteria, has been demonstrated in various models [53–55]. However, recent studies in *Drosophila* lacking the major lysozyme genes showed that the effect of intestinal lysozymes on endogenous gut microbiota was less pronounced than expected [47]. We found a similar result (i.e., that increased lysozyme expression did not lead to a massive decrease in bacterial load but rather to an increase; Fig. 5A). However, there was a shift in the composition of the microbiota. We chose two simple model bacteria species, the gram-negative *A. thailandicus* [48] and the gram-positive *L. plantarum* [56]. The increased lysozyme expression in the *dSirt4* knockout flies only had a minor effect on the microbial load but significantly changed the composition of this very simple model microbiome. A shift towards gram-negative bacteria was observed, caused by a reduction in the number of gram-positive bacteria but no change in the number of gram-negative bacteria. This shift was attributed to increased lysozyme expression because it was not observed in a lysozyme-deficient background. Therefore, there may be a direct mechanistic link between the lysozyme expression and the change in the microbiota composition. The observed effects of dSirt4 depletion on microbiota composition cannot be solely attributed to changes in lysozyme expression; other antimicrobial factors also play a crucial role. Notably, the altered abundance of antimicrobial peptides should be highlighted. Once again, predicting the effects of changing the microbiota composition on health-related parameters in *Drosophila is challenging*. The expected preference for *Lactobacillus* species may mediate both positive and negative effects. For instance, increased concentrations of *Lactobacillus plantarum* have been associated with improved intestinal barrier function [57]. However, a significant shift towards *Lactobacilli* could lead to adverse dysbiotic effects [58].

The complex role of lysozymes in the *Drosophila* intestine is mirrored in the mouse intestine. An imbalance in lysozyme expression in Paneth cells significantly affects the inflammatory tone of the intestine, which has implications for the development of chronic IBDs; this finding could significantly impact that field. Lysozyme 1 deficiency protects against inflammatory responses, whereas its ectopic overexpression promotes them [59]. Therefore, excessive lysozyme expression correlates with a dysbiotic situation [59]. A similar situation exists in *Drosophila*. This dysbiosis could explain the shortened lifespan of flies with *dSirt4* knocked out in enterocytes. The fact that we could show that both *dSirt4* knockout in enterocytes and the enterocyte-specific overexpression of lysozymes reduce lifespan supports this hypothesis.

Sirtuin activity strongly depends on ingested food and is generally associated with the health-promoting effects of caloric restriction [60, 61]. We demonstrated a similar effect in the *Drosophila* gut, where only *dSirt4* was upregulated, while other sirtuin genes were downregulated. Several factors other than nutrition may regulate sirtuin activity. First and foremost is infection with intracellular bacteria of the genus *Wolbachia*, which is known to regulate *dSirt4* expression and impact the microbiota [27]. The effects this has on lysozyme expression and the possible dysbiotic composition of the microbiota must be clarified in the future.

In addition to this exciting result—the increased level of lysozymes and the associated changes in the microbiota—*dSirt4* deficiency leads to other interesting changes in the gut. Notably, its fundamental metabolic properties, which are known to be associated with sirtuins [12, 62], have been reprogrammed. The effects on carbohydrate metabolism are significant, including a massive reduction in the expression of the target of brain insulin (*tobi*), a key regulator of carbohydrate metabolism [63]. The increased expression of many members of the maltase family and the lysosomal alpha-mannosidases supports this anticipated impact on carbohydrate metabolism. The increased expression of the latter in the gut extends lifespan [64], consistent with our results. We also found a reduced abundance of proteins involved in lipid transport processes, which may explain the reduced ability of flies to utilize fat reserves in response to starvation. The reduced abundance of apolipophorin (aplopp) and Niemann-Pick type C-1b (Npc1b) is critical [65, 66].

In summary, we have identified *dSirt4* as a highly sensitive cellular sensor in the adult *Drosophila* gut that responds to diet changes and broadly reprograms gut metabolism. Of particular note is the expression of lysozymes, which provides a direct mechanistic link to the microbiota composition and significantly advances our understanding of the development of chronic inflammatory diseases of the gut, where sirtuins may play an important role [17].

## Electronic supplementary material

Below is the link to the electronic supplementary material.

Supplementary Material 1: Tab. S1: dSirt4 KO Proteome Data Set, Enrichment Analyses and 1D Annotation Enrichment.

Supplementary Material 2: Fig. S1 (A) Changes in expression of sirtuins in response to DSS. All five sirtuins are upregulated in *w*^*1118*^ flies after 48 h of treatment with 5% DSS (*n* = 5). (B) Changes in sirtuin expression in response to starvation. *dSirt1* and *dSirt2* are downregulated after 24 h of starvation, while expression of other sirtuins is not affected (*n* = 4–5). (C) Increase of lysozyme activity in *dSirt1* KO and *dSirt2* KO intestines (*n* = 6–8). * = *p* < 0.05, ** = *p* < 0.01, **** = *p* < 0.0001. Fig. S2 Lysozyme counts in the different cell types of the adult intestine. Analysis was performed using the following entry: https://www.flyrnai.org/scRNA/gut/. Fig. S3 Relative abundance of the genus *Acetobacter* in the different conditions using 16 S rRNA gene sequencing. The pairwise Wilcoxon rank sum test was used to compare the abundances between conditions. P-values were corrected for multiple testing using the FDR correction method. *N* = 4 per condition, * = *p* < 0.05.

## Data Availability

The proteomic analysis results are provided in Supplementary Table S1. The MS data were deposited to the ProteomeXchange Consortium [36] by the PRIDE partner repository (dataset identifier: PXD054704 (access for reviewer only: Username: reviewer_pxd054704@ebi.ac.uk Password: O9RH0Xw5oR4O).
