# Supplementary material for: Mitochondrial sirtuin 4 shapes the intestinal microbiota of Drosophila by controlling lysozyme expression
Source: Anim Microbiome. 2025 Jun 13;7:63. doi: 10.1186/s42523-025-00431-x (PMC12166577; doi:10.1186/s42523-025-00431-x)
Supplement: Supplementary file 2 — Supplementary Material 2: Fig. S1 (A) Changes in expression of sirtuins in response to DSS. All five sirtuins are upregulated in w1118 flies after 48 h of treatment with 5% DSS (n = 5). (B) Changes in sirtuin expression in response to starvation. dSirt1 and dSirt2 are downregulated after 24 h of starvation, while expression of other sirtuins is not affected (n = 4–5). (C) Increase of lysozyme activity in dSirt1 KO and dSirt2 KO intestines (n = 6–8). * = p < 0.05, ** = p < 0.01, **** = p < 0.0001. Fig. S2 Lysozyme counts in the different cell types of the adult intestine. Analysis was performed using the following entry: https://www.flyrnai.org/scRNA/gut/. Fig. S3 Relative abundance of the genus Acetobacter in the different conditions using 16 S rRNA gene sequencing. The pairwise Wilcoxon rank sum test was used to compare the abundances between conditions. P-values were corrected for multiple testing using the FDR correction method. N = 4 per condition, * = p < 0.05. [file 42523_2025_431_MOESM2_ESM.docx]

**Supplementary figures**


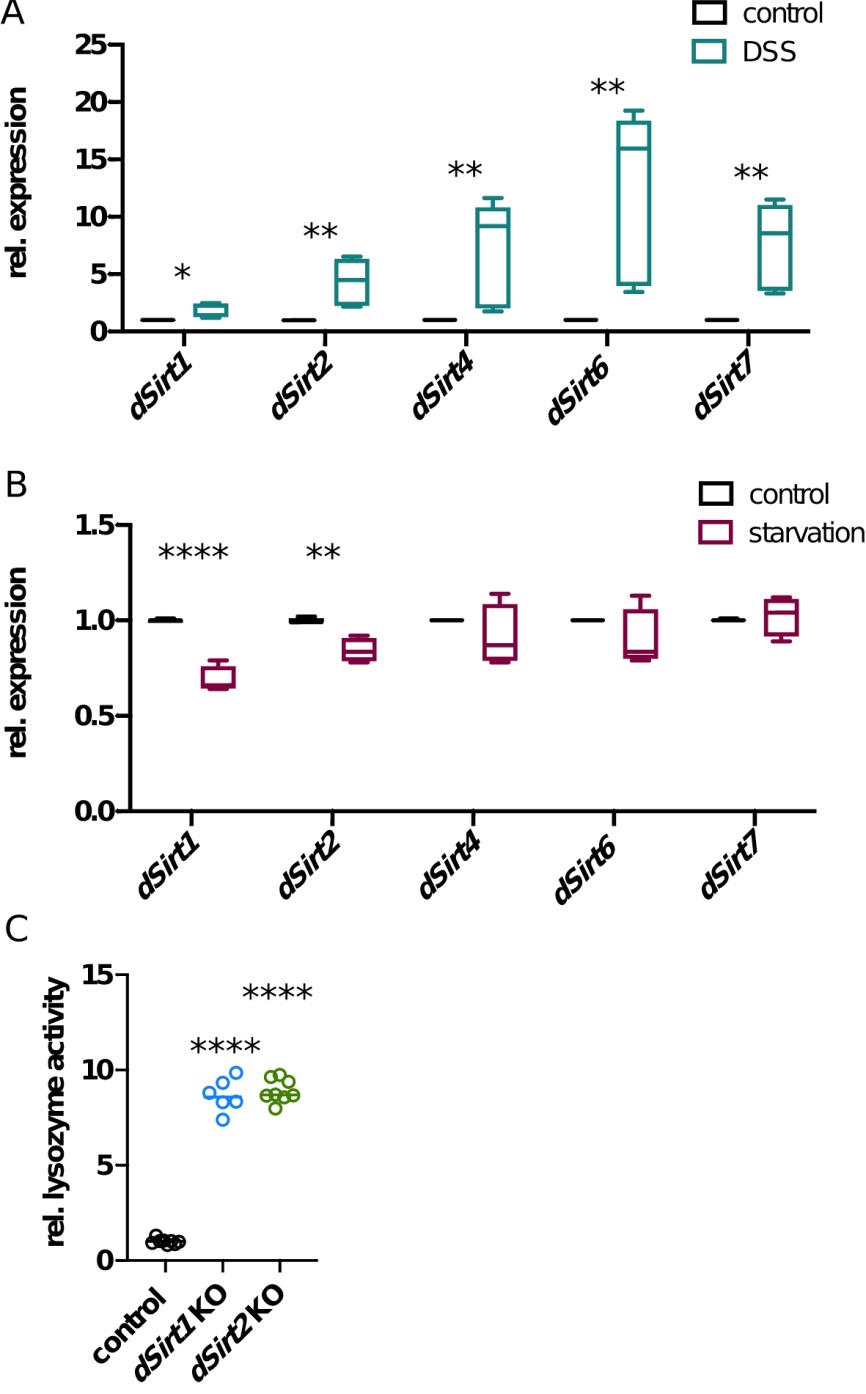


**Fig. S1:** (A) Changes in expression of sirtuins in response to DSS. All five sirtuins are upregulated in *w^1118^* flies after 48 h of treatment with 5% DSS (n = 5). (B) Changes in sirtuin expression in response to starvation. *dSirt1* and *dSirt2* are downregulated after 24 h of starvation, while expression of other sirtuins is not affected (n = 4–5). (C) Increase of lysozyme activity in *dSirt1* KO and *dSirt2* KO intestines (n = 6–8). * = *p* < 0.05, ** = *p* < 0.01, **** = *p* < 0.0001.


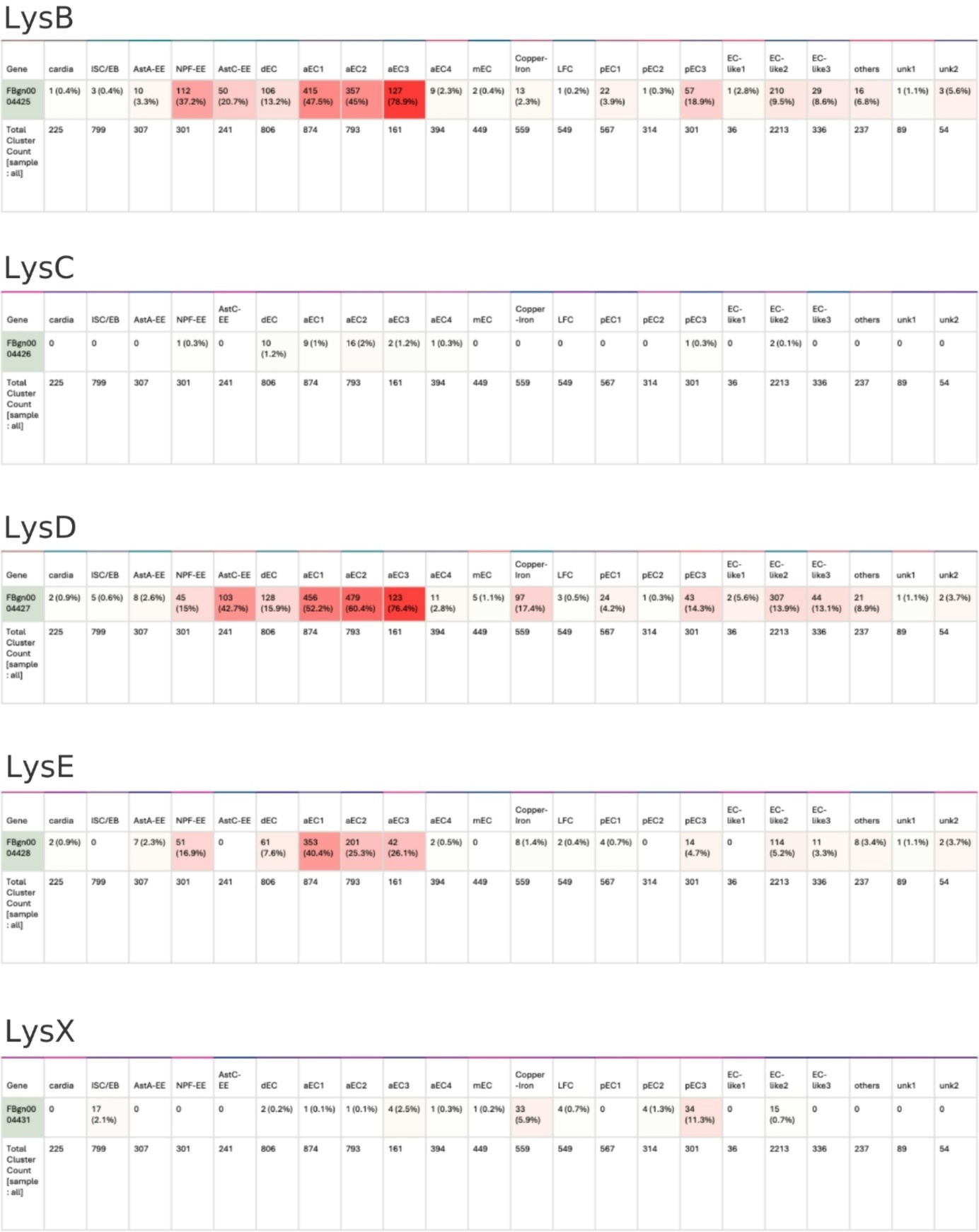



**Fig. S2:** Lysozyme counts in the different cell types of the adult intestine. Analysis was performed using the following entry: https://www.flyrnai.org/scRNA/gut/.


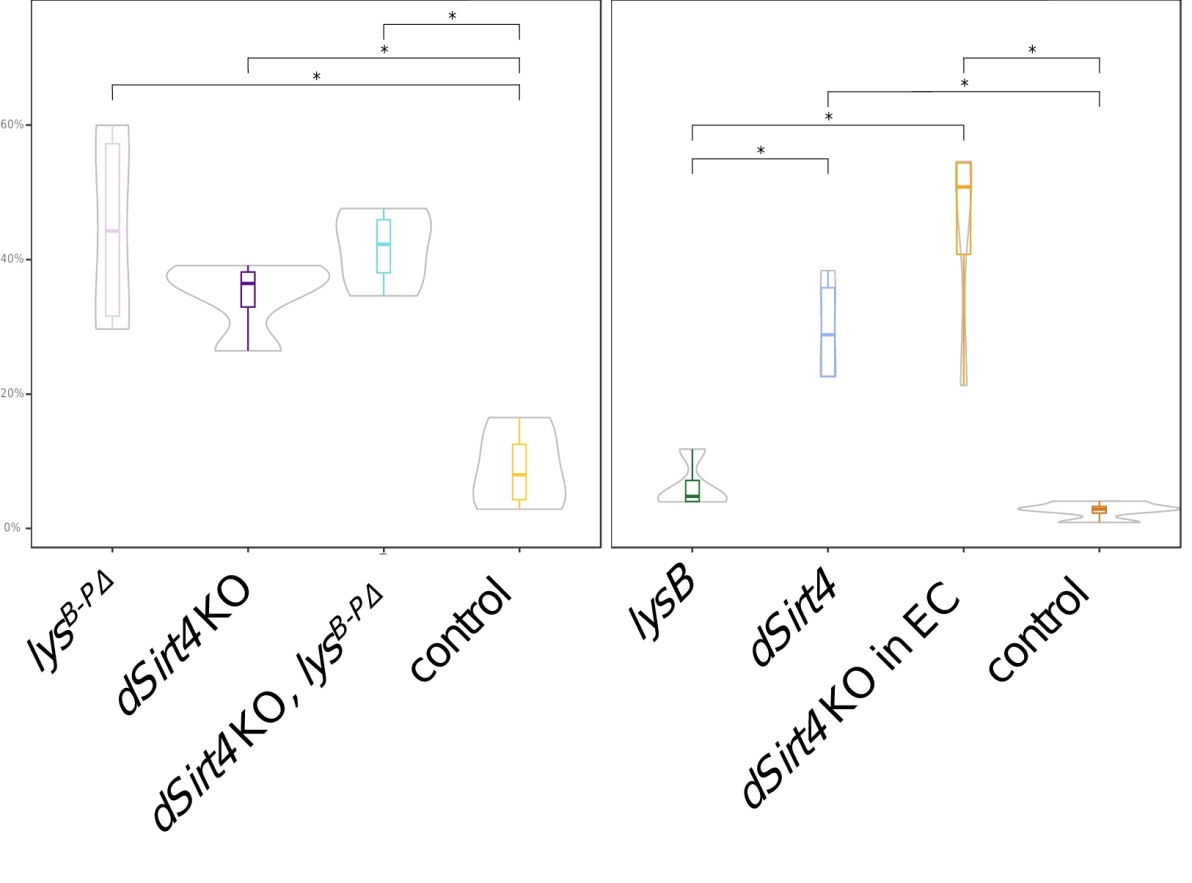


**Fig. S3:** Relative abundance of the genus *Acetobacter* in the different conditions using 16S rRNA gene sequencing. The pairwise Wilcoxon rank sum test was used to compare the abundances between conditions. P-values were corrected for multiple testing using the FDR correction method. N = 4 per condition, * = *p* < 0.05

**Supplementary table**

Tab. S1: dSirt4 KO Proteome Data Set, Enrichment Analyses and 1D Annotation Enrichment.
